# Dental Caries Detection in Children Using Intraoral Scanners Featuring Fluorescence: Diagnostic Agreement Study

**DOI:** 10.2196/78023

**Published:** 2025-12-08

**Authors:** Bree Jones, Tong Chen, Stavroula Michou, Nicky Kilpatrick, Nigel Curtis, David P Burgner, Christoph Vannahme, Mihiri Silva

**Affiliations:** 1 Melbourne Dental School, University of Melbourne, 720 Swanston Street, Carlton, 3035, Australia, 61 9341-4000; 2 Murdoch Children's Research Institute, Royal Children's Hospital, Parkville, Australia; 3 Clinical Epidemiology & Biostatistics Unit, Murdoch Children's Research Institute, Royal Children's Hospital, Parkville, Australia; 4 Department of Odontology, School of Health and Medical Sciences, University of Copenhagen, Nørre Allé, Denmark; 5 Department of Paediatrics, University of Melbourne, Royal Children's Hospital, Parkville, Australia; 6 Department of Infectious Diseases, Royal Children's Hospital, Parkville, Australia; 7 3Shape TRIOS A/S, Copenhagen, Denmark

**Keywords:** dental caries, dentition, primary, image interpretation, computer-assisted, models, dental, imaging, 3-dimensional, computer-assisted

## Abstract

**Background:**

Dental caries is a common chronic disease in children. Digital tools such as intraoral scanners (IOSs) may offer an efficient, scalable alternative to conventional visual examination for dental caries detection. IOSs are handheld devices that generate 3-dimensional (3D) models of the teeth and surrounding structures. Recent advances incorporating fluorescence technology into scanner hardware offer the potential for supporting dental caries detection. However, the performance of digital caries detection methods using 3D models that include both color and fluorescence in children’s teeth remains unknown.

**Objective:**

This study aimed to assess the diagnostic agreement between visual examination and on-screen assessment of 3D models generated by an IOS in color and supplemented with fluorescence for caries detection in primary teeth.

**Methods:**

Children participating in a clinical trial (n=216, mean age 5.6, SD 0.4 y) underwent visual examination using the International Caries Detection and Assessment System (ICDAS) and intraoral scanning using the TRIOS 4 IOS. Four trained registered dental practitioners independently assessed each participant’s 3D models in color and then supplemented with fluorescence, using a previously validated ICDAS index modified for on-screen assessments of 3D models. All 3D models were assessed again after 4 weeks. The time taken for intraoral scanning and on-screen assessment was recorded. Multilevel logistic regression was used to estimate and compare the likelihood of detecting caries between methods, and Bland-Altman plots were used to visualize agreement. Analyses were performed at the initial (ICDAS ≥01), moderate (ICDAS ≥03), and extensive (ICDAS ≥05) dental caries thresholds. Intraclass correlation coefficient (ICC) estimated method agreement and examiner reliability.

**Results:**

Of 219 children enrolled, 216 completed dental assessments. A total of 9470 visible primary tooth surfaces were included in the analysis. The average time taken for on-screen assessment of each 3D model (color with fluorescence) was 3.5 (SD 2.3) minutes. The likelihood of detecting caries using color assessment of 3D models was similar to visual examination at all disease thresholds: initial (odds ratio [OR] 1.1, 95% CI 1.0‐1.3), moderate (OR 0.9, 95% CI 0.7‐1.1), and extensive (OR 1.0, 95% CI 0.7‐1.3). When color assessments were supplemented with fluorescence, the likelihood of detecting caries was 30% higher at the initial threshold relative to visual examination (OR 1.3, 95% CI 1.1‐1.5) and similar at the moderate (OR 0.9, 95% CI 0.7‐1.1) and extensive thresholds (OR 0.9, 95% CI 0.7‐1.3). Bland-Altman plots showed a high level of agreement at both moderate and extensive thresholds. Agreement between methods was high (ICC 0.9, 95% CI 0.9‐1.0). Intra- and inter-examiner reliability using intraoral scans ranged from good to excellent (ICC 0.8‐1.0).

**Conclusions:**

On-screen assessment of 3D models in color demonstrated the highest agreement with visual examination for caries detection across all disease thresholds.

## Introduction

Dental caries is a chronic, multifactorial disease influenced by environmental and behavioral factors [1]. It is estimated to impact more than half a billion children globally, representing a major public health concern [2]. Developing efficient and accurate tools to collect data on dental caries can facilitate the inclusion of oral health outcomes in epidemiological research, improving understanding of contemporary risk factors, disease distribution, and the burden of disease in children.

The clinical presentation of dental caries spans a spectrum from early to advanced disease. In its earliest stage, mineral loss causes subsurface changes that alter enamel porosity, and subsequently its optical properties. At this point, the enamel surface remains intact, and the process can be reversed or arrested through remineralization strategies [3]. If mineral loss continues, tooth structure becomes compromised, potentially leading to cavity formation. These later stages signify irreversible damage to the tooth and may eventually result in pain and tooth loss if left untreated [3].

Conventional assessment of dental caries in epidemiological research involves a visual examination by a qualified dental practitioner using a validated scoring index. Implementing visual examinations across large or multisite studies requires substantial investment in examiner training and calibration to ensure consistency, as interexaminer variability can impact study validity [4]. The financial and logistical challenges may limit the use of visual examinations, with some studies instead relying on self-reported caries status. Self-reporting methods have been criticized for their lack of validity and their tendency to underestimate disease burden in children [5].

Intraoral scanners (IOSs) are hand-held devices that generate 3-dimensional (3D) digital scans of the teeth and surrounding structures (herein 3D models). It has been suggested that 3D models can be used to perform digital dental examinations [6]. The various commercially available devices differ in their hardware and software for image acquisition and reconstruction. Manufacturers include, but are not limited to, Align Technologies (San Jose, California, USA), Medit Corp (Seoul, South Korea), Dentsply Sirona (Charlotte, NC, USA), Planmeca (Helsinki, Finland), and 3/Shape (Copenhagen, Denmark).

The TRIOS 4 and TRIOS 5 scanners (3/Shape, Copenhagen, Denmark) incorporate light-induced fluorescence technology into their hardware to aid dental caries detection. The devices use an LED light source (approximately 415 nm) and a long-pass filter to capture emitted red and green fluorescence [7]. When healthy teeth are exposed to blue light, they become excited to emit green fluorescence [8]. Areas affected by dental caries have reduced green fluorescence intensity, as mineral loss alters the tooth's composition and its light-scattering properties[9] At these wavelengths, some bacterial metabolites associated with dental caries emit red fluorescence. Using the scanner software, green and red fluorescence can be visualized simultaneously as an overlay on the 3D model. Fluorescence characteristics can be interpreted by a dental practitioner to support detection of subtle early lesions which may be difficult to visualize when viewing scans in color alone.

The International Caries Detection and Assessment System is a widely accepted scoring index for detecting and classifying dental caries severity [10]. To reduce subjectivity, a modified version incorporating fluorescence features has been developed [11] and validated [12]. In adult permanent teeth, an in vitro study investigating on-screen assessment using this index found no difference in diagnostic accuracy between on-screen assessment and conventional visual examination for occlusal tooth surfaces [13]. However, the real-world diagnostic performance of an on-screen caries assessment for primary teeth and other surface types (such as visible smooth surfaces) is unknown.

Clinically, enamel defects represent another prevalent condition that can resemble dental caries, as affected areas often display similar visual characteristics. There is also overlap in the indices used to record and quantify both conditions [14]. Yet, most studies evaluating caries detection methods typically exclude teeth with enamel defects, leaving their influence on diagnostic performance uncertain.

This study, therefore, aimed to determine the in vivo diagnostic agreement between visual examination and on-screen assessment of 3D models in approximate natural colors with and without fluorescence for detecting and classifying caries in primary teeth. The secondary aims included exploring the impact of disease threshold, enamel defects, and surface type on agreement estimates.

## Methods

### Study Design

**Figure 1.**
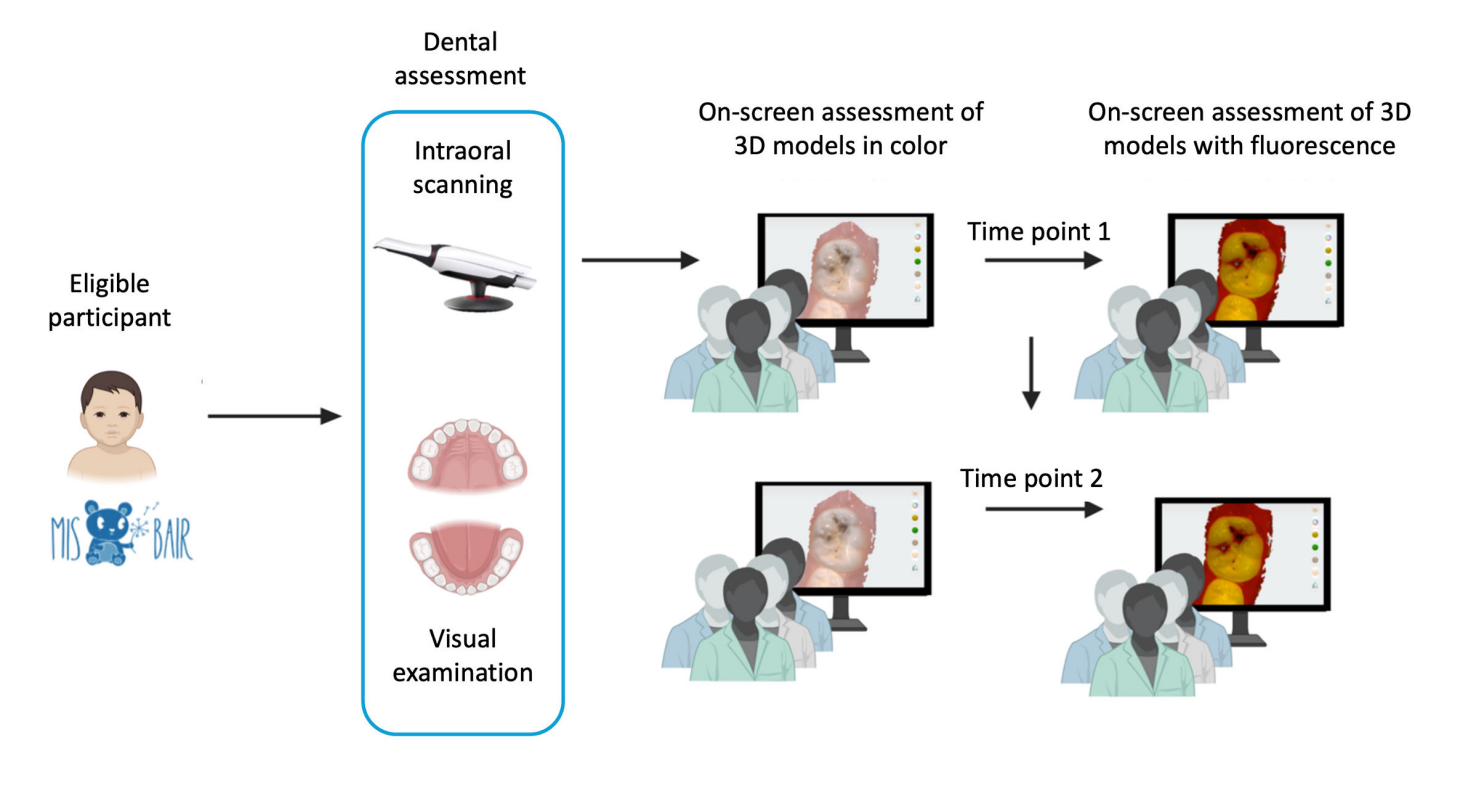
Study design schema: study design schema illustrating the cross-sectional diagnostic agreement study nested within the MIS BAIR randomized controlled trial. The study compared the diagnostic agreement for dental caries detection and classification between visual examination and 2 on-screen assessment modalities (3D color models and 3D color with fluorescence models). Four trained examiners independently completed on-screen assessments at 2 time points (>4 wk apart). Visual examination outcomes served as the comparator. This image is created using BioRender [18].

A cross-sectional diagnostic agreement study was undertaken (Figure 1), nested within a longitudinal randomized controlled trial. An agreement design was selected as the visual examination comparator was not considered a gold standard reference in the study setting [15]. This study followed the Guidelines for Reporting Reliability and Agreement Studies [16] (Table SA-1 in Multimedia Appendix 1) and was conducted per the applicable sections of the Standards for Reporting of Diagnostic Accuracy Studies [17]. The protocol was prospectively published (International Registered Report Identifier DERR1-10.2196/51578) [15] and registered with the Australian and New Zealand Clinical Trials Registry (ID ACTRN12622001237774).

### Study Setting and Participants

The subjects were participants in the Melbourne Infant Study: BCG for Allergy and Infection Reduction (MIS BAIR), a phase III multicenter, randomized controlled trial [19]. The trial aimed to investigate the impact of neonatal BCG vaccination on infection and allergy outcomes in early childhood. Eligible English-speaking mothers in Victoria (2013‐2016) consented for their newborns to join MIS BAIR. Infants with contraindications to live vaccines at birth were excluded, yielding 1272 participants. Clinical assessments were conducted at 1 year and 5 years.

Trial participants received monthly newsletters and accessed study updates via the MIS BAIR website, where families were informed of an optional dental assessment (which included intraoral scanning) at the 5-year-old follow-up. The dental component commenced in January 2021, with 536 participants remaining in the trial. Despite COVID-19 disruptions, visits continued under special approval, and 219 participants had consented to dental assessments by March 2022. All dental assessments were performed at Murdoch Children’s Research Institute (Melbourne, Australia).

All primary coronal tooth surfaces were eligible for inclusion in the agreement study if they were examined during the clinical visual examination and were visible for on-screen assessment on the corresponding intraoral scan. Tooth surfaces were excluded if they were located on proximal aspects, restored, sealed, had missing or insufficient scan data, or were not visible due to other reasons.

### Dental Assessment Procedure

Visual examination was performed with participants in a semisupine position, under illumination, using a dental mirror and probe with no magnification. Two trained and calibrated dental practitioners examined all teeth after removing gross plaque deposits using Puritan swabs and drying teeth with cotton wool rolls and gauze. Training procedures are described elsewhere [15]. Universal infection control standards and additional COVID-19 precautions were adhered to for all examinations.

All tooth surfaces were examined following a standard operating procedure. Any reasons for noncompletion of any parts of the dental examination were recorded. Dental caries was recorded using a merged International Caries Detection and Assessment System (ICDAS) criteria [11], and enamel defects were recorded using a modified version of the European Academy of Paediatric Dentistry index [20]. If there were multiple lesions present on the one tooth surface, the most severe of the 2 clinical presentations was recorded.

**Figure 2.**
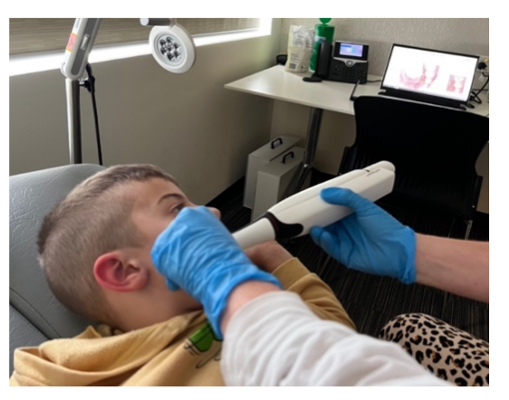
Intraoral scanning procedure. A participant undergoing intraoral scanning as part of this research. The participant can observe the intraoral scan being constructed in real time during the procedure.

Teeth were scanned with the TRIOS 4 IOS immediately following the visual examination. The laptop was set up on a table across from the participant, where they were able to view scan acquisition during scanning (Figure 2). A scan was deemed adequate when sufficient color and fluorescence data were captured. Incomplete scans were documented, with common causes, including scanner tip access, gag reflex, or limited cooperation with scanning due to behavioral or developmental factors. Each scan was saved under a unique identifier using the TRIOS Dental Desktop software with the TRIOS module. The scanning time was calculated using the TRIOS software. The IOSs were regularly calibrated according to the manufacturer’s procedures. The manufacturer scanning strategies were followed, and external light was minimized during scanning.

### On-Screen Assessment Procedure

**Figure 3.**
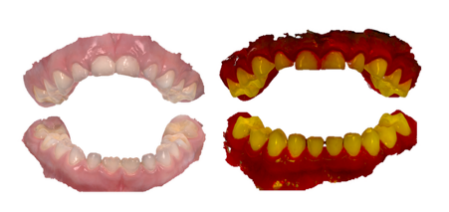
3D models generated by an intraoral scanner. Left: Approximate natural color textures. Right: With the addition of fluorescence texture.

On-screen assessments of 3D models commenced in November 2022, 8 months after the dental assessments. Examiner training and calibration processes for on-screen assessments are detailed in the study protocol [15] and Table SA-2 in Multimedia Appendix 1). Assessments were performed on 15-inch monitors using Windows 11 in rooms with natural lighting. A custom noncommercial software (developed by 3Shape) was used to efficiently retrieve and view multiple postprocessed 3D models at resolution quality equivalent to TRIOS Dental Desktop. A systematic visual examination protocol guided all on-screen assessments. Four trained dental examiners, blinded to the clinical data and each other’s ratings, independently evaluated each participant’s models in 2 stages: first assessing surface geometry in approximate natural colors and then supplemented with fluorescence data (Figure 3). The presence and extent of dental carious lesions were recorded using a validated, modified ICDAS index incorporating fluorescence descriptors (Figure SA-1, Multimedia Appendix 1) [11-13,15]. The models were assessed at time point 1. All examiners repeated the on-screen assessment protocol >4 weeks later (time point 2).

All data were recorded and managed using a secure, web-based app, the REDCap (Research Electronic Data Capture) tool [21].

### Outcome Measures

The primary outcome was dental caries at a tooth surface level. Three binary outcome variables were derived to represent clinically relevant disease thresholds for dental caries. Specifically, caries data were dichotomized at initial (ICDAS ≥01), moderate (ICDAS ≥03), and extensive disease thresholds (ICDAS ≥05). All other variable definitions are described in Table SA-3 (Multimedia Appendix 1).

### Sample Size

The sample was drawn from all 219 eligible children who participated in the MIS BAIR dental assessment at the 5-year-old follow-up visit.

### Statistical Analysis

Evaluating tooth surface-level outcomes in dental research introduces complexities to the statistical analysis and limits the use of traditional methods for examining diagnostic agreement. Whole mouth dental data have multiple levels of clustering (surfaces nested within a tooth and teeth nested within an individual), and the high prevalence of healthy tooth surfaces results in a zero-inflated data distribution.

First, to account for the clustering, this study used multilevel logistic regression to estimate the likelihood of caries detection at the tooth surface level. The likelihood of detecting dental caries between visual examination and on-screen assessment methods (with and without fluorescence) was estimated by including an indicator variable for the caries detection method as a fixed effect. Additionally, the 2 on-screen assessment methods were compared by fitting a separate model that included method, rater, and time (time point 1 or time point 2) as fixed effects. A sensitivity analysis was performed by adjusting the model for enamel defects, and an additional sensitivity analysis by surface type (smooth surfaces vs occlusal surfaces) investigated if estimates differed based on surface location.

Second, the agreement between methods for the total number of caries detected for each participant was examined using the Bland-Altman plot [22]. The Bland-Altman plot compared the differences in the total number of carious surfaces detected between methods (y-axis) against the mean of the 2 methods (x-axis) for each subject. Due to the significant overlap of points, the size of the points in the plot was adjusted to reflect the frequency of the observations. Visual examination was compared to on-screen assessment with and without fluorescence at each diagnostic threshold (initial, moderate, and extensive). This comparison was repeated for each surface type (occlusal and smooth surfaces). A best-fit regression line of the differences in methods against the mean was plotted to identify trends in differences across the range of averages.

Finally, the intraclass correlation coefficient (ICC) was calculated to estimate the concordance between visual examination and on-screen assessment methods (with and without fluorescence) for total carious surfaces detected by each method per participant. The ICC for count data was estimated from generalized linear mixed models with zero-inflated Poisson distribution to account for the excess zero observations [23]. For the primary method comparison, given the multiple examiner observations for the on-screen assessment methods, the median number of surfaces with carious lesions detected among examiners for each participant was used to compare to the visual examination. Additionally, the ICC was calculated for each combination of raters to determine the concordance (interrater reliability) for on-screen assessments (with and without fluorescence) and the repeatability (intrarater reliability) for the same rater between time point 1 and time point 2 for on-screen assessments. All analyses were performed in STATA version 18 (StataCorp) [24] and R (R Core Team) [25].

### Ethical Considerations

This research was conducted with the approval of the Royal Children’s Hospital Human Research Ethics Committee (RCH HREC No.88321). Written parental or guardian consent was obtained for each participant to allow inclusion in the study and the use of dental data for validating and advancing intraoral scanning technology for oral health assessment. Children received a AUD $20 gift card (US $15) as remuneration for their participation. All participant records were assigned unique identifiers, ensuring that individual identities were not identifiable by the dental team. The study was conducted according to the principles outlined in the Declaration of Helsinki and the ICMJE recommendations. The study protocol is registered with the Australian New Zealand Clinical Trials Registry (registration number: ACTRN12622001237774p). The study protocol was published a priori with the International Registered Report Identifier DERR1-10.2196/51578. The MIS BAIR randomized controlled trial had ethical and governance approval from Mercy Health Human Research Ethics Committee (HREC; No. R12-28) and Royal Children’s Hospital HREC (No. 33025).

## Results

### Study Flow

**Figure 4.**
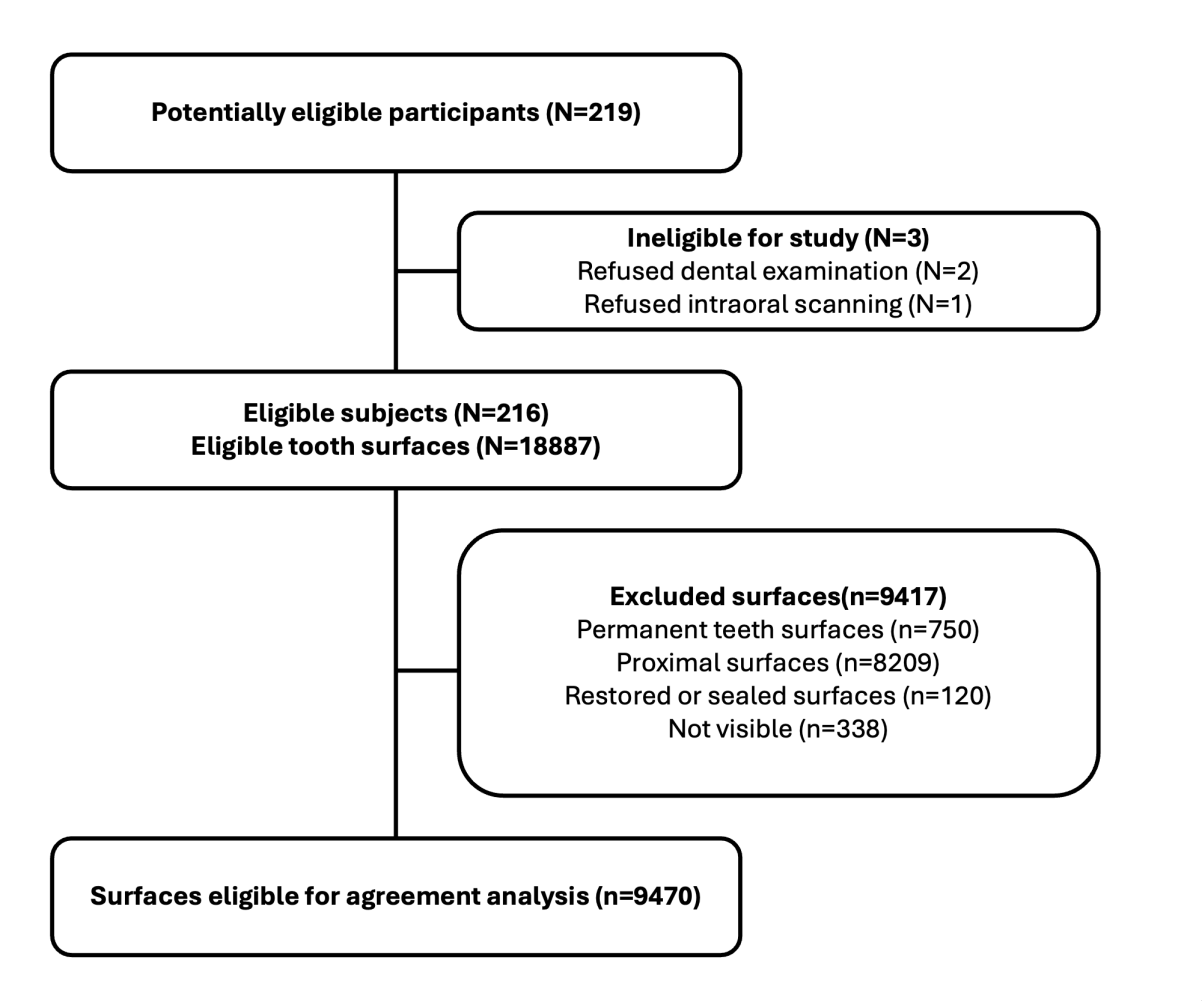
Study flow diagram. Flow diagram outlining patient eligibility and exclusion criteria (N=number of patients, n=number of tooth surfaces).

Of the 219 children who consented to dental assessments, 3 declined dental examinations or intraoral scanning at the time of the study visit, leaving a study population of 216. A total of 18,887 tooth surfaces were eligible for inclusion. Permanent tooth surfaces (n=750), proximal tooth surfaces (n=8209), restored or sealed tooth surfaces (n=120), and surfaces that were not visible or that could not be examined due to incomplete or missing scan data (n=338) were excluded. The remaining 9470 (50%) tooth surfaces were included in the analysis (Figure 4).

### Sample Characteristics

Table 1 presents the characteristics of the sample. Participants’ average age was 5.6 (SD 0.4) years, with 49% males and 51% females. Most children had mothers of Anglo-Celtic ancestry (n=124, 57%), followed by European (n=38, 18%) and Asian (n=33, 15%). Most children resided in the state’s most advantaged areas (n=138, 64%). On the basis of visual examination data, the prevalence of dental caries at the initial disease threshold was 38%, and enamel defects were 18%.

**Table 1.** Sample characteristics (N=216).

Participant characteristics	Values
Age (y), mean (SD)	5.6 (0.4)
Sex, n (%)	
Male	106 (49.1)
Female	110 (50.9)
Ethnicity, n (%)	
Anglo-Celtic	124 (57.4)
European	38 (17.6)
Asian	33 (15.3)
Middle East	4 (1.9)
African	4 (1.9)
Aboriginal/Torres Strait Islander	2 (0.9)
Other	11 (5.1)
SEIFA^a^, n (%)	
Quintile 1	5 (2.3)
Quintile 2	31 (14.4)
Quintile 3	42 (19.4)
Quintile 4	49 (22.7)
Quintile 5	89 (41.2)
Prevalence of caries	82 (37.9)
Prevalence of enamel defects	39 (18.1)

a Socio-economic Indexes for Areas (SEIFA) ranks areas in Australia according to relative socioeconomic disadvantage. SEIFA was divided into quintiles ranging from the most disadvantaged (quintile 1) to the least disadvantaged (quintile 5).

### Examiner Characteristics

Four registered dental practitioners undertook on-screen assessments for all participants at 2 time points. They were recruited via convenience sampling. The examiners had clinical experience ranging from 5 to 15 years, including conducting epidemiological assessments (Table SA-2 in Multimedia Appendix 1).

### Time Taken for Scanning and On-Screen Assessments

The mean time to perform intraoral scanning of the lower arch was 46 (SD 8) seconds and for the upper arch was 58 (SD 15) seconds. The mean time taken for the on-screen assessment of each 3D model in color supplemented with fluorescence was 3.5 (SD 2.3) minutes.

### Surface-Level Agreement

The total number of carious tooth surfaces reported for each method at each diagnostic threshold and odds ratios (ORs) are presented in Table 2, Table 3, and Table 4 (Raw data stratified by rater and surface type are available in Table SA-4 and Table SA-5 Multimedia Appendix 1). The likelihood of detecting caries using on-screen assessment of 3D models in color was similar to that of visual examination at the initial (odds ratio [OR] 1.1, 95% CI 1.0‐1.3), moderate (OR 0.9, 95% CI 0.7‐1.1), and extensive (OR 1.0, 95% CI 0.7‐1.3) disease thresholds. When 3D models were supplemented with fluorescence, the likelihood of detecting caries was 30% higher at the initial threshold relative to visual examination (OR 1.3, 95% CI 1.1‐1.5) and similar at the moderate (OR 0.9, 95% CI 0.7‐1.1) and extensive thresholds (OR 0.9, 95% CI 0.7‐1.3). The sensitivity analysis by surface type is also presented in Table SA-6 in Multimedia Appendix 1. The likelihood of caries detection using on-screen assessment with and without fluorescence was comparable to visual examination for smooth surfaces across all disease thresholds. For occlusal surfaces, the likelihood of reporting caries with on-screen assessment methods was 1.5- to 2-fold higher at the initial disease threshold both with (OR 2.0, 95% CI 1.6‐2.4) and without fluorescence (OR 1.5, 95% CI 1.2‐1.9). The likelihood of reporting at the moderate and extensive thresholds on occlusal surfaces was similar for on-screen assessments and visual examinations. Comparing on-screen assessment methods reveals that adding fluorescence increases the likelihood of caries detection, and this likelihood is also influenced by the rater scoring the assessment. Accounting for enamel defects as an additional covariate in the analysis resulted in slightly lower OR estimates for both methods across all disease thresholds.

**Table 2.** Agreement estimates for visual examination (VE) versus on-screen assessment (OSA) for all included tooth surfaces (n=9470).

Disease threshold	VE		OSA		Main analysis^a^		Sensitivity analysis	
	No	Yes	No	Yes	OR (95% CI)	*P* value	OR (95% CI)	*P* value
Initial threshold	9182	288	9154	316	1.1 (1.0-1.3)	0.08	1.1 (1.0-1.3)	0.03
Moderate threshold	9384	86	9395	75	0.9 (0.7-1.1)	0.19	0.8 (0.7-1.1)	0.17
Extensive threshold	9422	48	9424	46	1.0 (0.7-1.3)	0.78	1.0 (0.7-1.3)	0.76

a The main analysis, which contained the variable Method as a fixed effect, is shown in addition to the sensitivity analysis, where enamel defects at VE were fit as an additional covariate in the multilevel model.

**Table 3.** Agreement estimates for visual examination (VE) versus on-screen assessment with fluorescence (OSA-FLU) for all included tooth surfaces (n=9470).

Disease threshold	VE		OSA- FLU		Main analysis^a^		Sensitivity analysis	
	No	Yes	No	Yes	OR (95% CI)	*P* value	OR (95% CI)	*P* value
Initial threshold	9182	288	9125	345	1.3 (1.1-1.5)	<.001	1.2 (1.1-1.4)	<.001
Moderate threshold	9384	86	9394	76	0.9 (0.7-1.1)	0.24	0.9 (0.7-1.0)	0.21
Extensive threshold	9422	48	9380	45	0.9 (0.7-1.3)	0.61	0.9 (0.7-1.3)	0.59

a The main analysis, which contained the variable Method as a fixed effect, is shown in addition to the sensitivity analysis, where enamel defects at VE were fit as an additional covariate in the multilevel model.

**Table 4.** Agreement estimates for on-screen assessment (OSA) versus on-screen assessment with fluorescence (OSA-FLU) for all included tooth surfaces (n=9470).

Disease threshold	OSA		OSA- FLU		Main analysis^a^		Sensitivity analysis	
	No	Yes	No	Yes	OR (95% CI)	*P* value	OR (95% CI)	*P* value
Initial threshold	9154	316	9125	345	1.1 (1.1-1.2)	<.001	1.1 (1.1-1.2)^b^	<.001
Moderate threshold	9395	75	9394	76	1.0 (0.9-1.2)	0.77	1.0 (0.9-1.2)^b^	0.77
Extensive threshold	9424	46	9380	45	1.0 (0.8-1.1)	0.62	1.0 (0.8-1.1)	0.62

a The main analysis, which contained the variable Method as a fixed effect, is shown in addition to the sensitivity analysis, where enamel defects at VE were fit as an additional covariate in the multilevel model.

b The inclusion of a rater variable in this model had a significant effect.

### Person-Level Agreement

**Figure 5.**
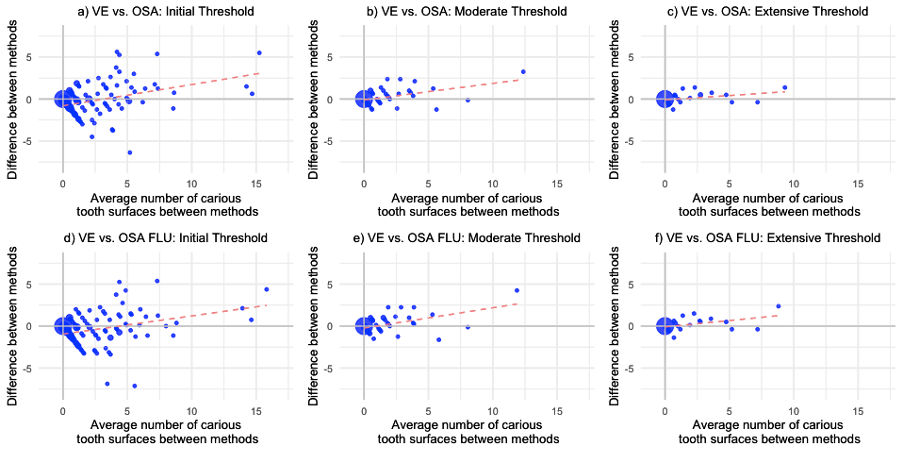
Bland-Altman plots of the differences between the methods for all tooth surfaces. Bland-Altman plots comparing the differences in the number of carious lesions detected for each pair of methods against the average of the 2 methods across the initial, moderate, and extensive disease thresholds. The regression line of the differences in methods against the mean is represented by a red dash. The size of the data points is proportional to the number of observations. (A) Initial threshold, OSA versus VE: absolute agreement n=67 (31%), negative differences n=108 (50%), positive differences n=41 (19%). (B) Moderate threshold, OSA versus VE: absolute agreement n=170 (79%), negative differences n=26 (12%), and positive difference n=20 (9%). (C) Extensive threshold, OSA versus VE: absolute agreement n=192 (89%), negative differences n=14 (6%), positive difference n=10 (5%). (D) Initial threshold, OSA-FLU versus VE: absolute agreement n=64 (30%), negative differences n=112 (52%), positive differences n=40 (18%). (E) Moderate threshold, OSA-FLU versus VE: absolute agreement n=167 (77%), negative differences n=28 (13%), positive differences n=21 (10%). (F) Extensive threshold, OSA-FLU versus VE: absolute agreement n=192 (89%), negative differences n=14 (6%), positive difference n=10 (5%). VE: visual examination; OSA: on-screen assessment in color only; OSA FLU: on-screen assessment with fluorescence.

The Bland-Altman plots indicate the absolute method agreement (Figure 5). For on-screen assessment of 3D models without (OSA) and with fluorescence (OSA-FLU) compared to visual examination, the absolute agreement was lower at the initial disease threshold and higher at the moderate and extensive disease thresholds. At the initial disease threshold, there were more negative differences compared to positive differences, where on-screen assessment recorded more carious surfaces relative to the visual examination (Figure 4A and D). At the moderate and extensive thresholds, the frequencies of positive and negative differences were comparable for both color-only and fluorescence-assisted assessments (Figure 4B, C, E, and F). The slope and intersection of the regression line, particularly at the initial caries thresholds, suggest that when average total carious surfaces were low, there were negative differences between methods. As the average total carious surfaces increased, the positive difference between methods increased (where visual examination recorded more carious surfaces relative to on-screen assessment). The subanalysis based on surface type shows similar trends (Figure SA-2 in Multimedia Appendix 1).

Table 5 presents the ICC values as a measure of method agreement and examiner reliability. On-screen assessment in color (ICC 0.9, 95% CI 0.9‐0.9) and with fluorescence (ICC 0.9, 95% CI 0.8‐0.9) showed good to excellent agreement with visual examination. For all examiners, the intrarater agreement of ICC of approximately >0.9 indicated a very high degree of scoring consistency within raters for on-screen assessment methods. The interrater reliability was also high between all raters (>0.9), indicating high-scoring similarity between raters. The narrow CIs further support the reliability of these estimates.

**Table 5.** Intraclass correlation coefficients (ICC). The ICC was used to estimate the absolute agreement between methods, intraexaminer reliability for on-screen assessment methods, and interrater agreement reliability for on-screen assessment methods.

	On-screen assessment with color, ICC (95% CI)	On-screen assessment with color and fluorescence, ICC (95% CI)
Agreement between methods		
Visual examination	0.9 (0.9-1.0)	0.9 (0.8-1.0)
Intrarater reliability		
Rater 1	0.9 (0.9-1.0)	0.9 (0.9-1.0)
Rater 2	0.9 (0.9-1.0)	0.9 (0.9-1.0)
Rater 3	0.9 (0.9-1.0)	0.9 (0.9-1.0)
Rater 4	0.9 (0.9-1.0)	0.9 (0.9-1.0)
Interrater reliability		
Rater 1 versus rater 2	0.9 (0.9-1.0)	0.9 (0.9-1.0)
Rater 1 versus rater 3	0.9 (0.9-1.0)	0.9 (0.9-1.0)
Rater 1 versus rater 4	0.9 (0.8-0.9)	0.9 (0.8-0.9)
Rater 2 versus rater 3	0.9 (0.9-1.0)	0.9 (0.9-1.0)
Rater 2 versus rater 4	0.9 (0.8-0.9)	0.9 (0.9-0.9)
Rater 3 versus rater 4	0.9 (0.8-0.9)	0.9 (0.8-0.9)

## Discussion

### Principal Findings

This study is the first to describe the real-world performance of IOSs using light-induced fluorescence for digital caries assessments of primary teeth in children. The findings suggest that the on-screen assessment of 3D models in color supplemented with fluorescence had a high level of agreement with visual examination for detecting primary coronal carious lesions on smooth and occlusal surface types at the moderate and extensive disease thresholds. At the initial disease thresholds, on-screen assessments resulted in a higher reporting of occlusal caries relative to visual examination, and supplementing on-screen color assessments with fluorescence increased the likelihood of dental caries detection compared to visual examination. The high ICC values provide further evidence of method agreement and indicate that the on-screen assessment criteria can be applied consistently and are reproducible.

Our findings are consistent with data from previous studies in adults. Porumb et al [26] observed high rater reliability and agreement between visual examination using ICDAS and on-screen assessment of 3D color models in adults when experienced examiners were used. Building on the work of a small pilot study that suggested remote assessment of 3D models in adults had good accuracy for cavity detection [6], our analysis shows that when applying a validated scoring index with trained examiners, assessment of intraoral scans has substantial agreement with visual examination. Our study results also corroborate the findings of several studies investigating on-screen assessments for permanent occlusal surfaces [12,13], showing that on-screen assessments had similar diagnostic performance to visual examinations. However, in contrast to these studies, our findings at the initial disease threshold, showed adding fluorescence increased the likelihood of reporting dental caries for occlusal surfaces.

The results of our study align with findings from prior research using intraoral cameras for dental caries data collection in pediatric epidemiological settings. In 5-year-old children, Boye et al [27,28] found the on-screen assessment of 2D images produced with intraoral cameras was equivalent to visual examination, although they used advanced disease thresholds (cavity involving dentin) for caries reporting. A study by Mehta et al [29] involving Australian 5-year-old children found that the on-screen assessment of 2D images resulted in significantly greater reporting of dental caries compared to visual examination when initial disease thresholds were applied, which the authors attributed to the improved lighting and examination conditions when performing on-screen assessments.

In the absence of a gold standard reference, it is not possible to determine whether the observed differences between methods reflect greater accuracy of one method over the other. The higher likelihood of reporting dental caries when on-screen assessment was supplemented with fluorescence could be attributable to several factors. It is plausible that on-screen assessment is more sensitive to early mineral loss than visual examination due to enhanced detection capabilities of light-induced fluorescence [30]. However, the apparent increase in detection may also reflect a reduction in specificity, as red fluorescence changes may be attributed to the presence of plaque leading to false-positive indications, particularly for occlusal surfaces, which often have plaque retentive pits and fissures [31]. The latter is consistent with findings investigating caries detection using 2D image-based ICDAS methods, which have reported lower specificity at the initial disease threshold [32]. The inherent subjectivity of early occlusal caries detection may also have contributed to the higher disagreements observed in our study at the initial disease threshold. Additionally, the creamy-colored demarcations associated with hypomineralized enamel could be misinterpreted as caries during the on-screen assessment, consistent with the reduced odds ratio estimates when enamel defects were included as a covariate in our sensitivity analysis.

### Strengths

The use of intraoral scanning for data collection in research offers several advantages. First, it enables examiner blinding to participant characteristics and allows repeat examinations, both of which improve internal consistency and reliability. Intraoral scanning may also facilitate more comprehensive dental assessments for children who can tolerate only brief or limited inspections. The resulting 3D models can be enlarged, rotated, and reviewed for extended periods, providing access to areas that may be difficult to view during visual examination alone. Furthermore, 3D models can serve as effective communication tools. A randomized controlled trial reported that 3D models as a visual aid significantly improved parents’ understanding of their children’s dental findings and treatment plans in pediatric dentistry compared to verbal explanations alone [33]. Finally, the data acquisition and on-screen assessment times achieved in this study were shorter than those reported in previous research using intraoral cameras for epidemiological research, which ranged from 6‐ to 8.8 minutes for data acquisition per participant [28].

### Limitations

We acknowledge several limitations. First, the time taken to undertake each dental examination was not recorded for all participants. Future studies should capture this, as it helps contextualize the clinical relevance. Second, the examination site lacked compressed air, and teeth were not professionally cleaned before examination and scanning. Third, unlike on-screen assessment, we did not undertake repeated visual examinations by multiple examiners. This is a potential source of bias, as it impacts the reliability of the visual examination as the comparator. Fourth, the highly selected sample potentially limits the broader generalizability of the findings. Additionally, a larger sample would increase the precision around the odds ratio estimates. Fifth, the study used an agreement design, which, although appropriate for this investigation, limits interpretation to relative rather than absolute diagnostic performance, as no gold standard reference was available. Sixth, dental caries vary in clinical presentation on proximal surfaces and around restorations or sealants, which were excluded from the analysis due to a lack of radiographic imaging in the study design. Given the age and disease experience of the cohort, it is not known how missing teeth, widened interdental spaces, recession, or heavily restored teeth may impact on-screen assessment performance. Further validation research in different population groups with varying levels of disease is warranted. Finally, the nonnormal distribution of the dataset precluded the calculation of Bland-Altman Limits of Agreement, which define an acceptable disagreement range among methods. Data transformations to address this lacked clinical interpretability. Clinically acceptable limits of agreement were therefore not defined, as establishing these would require a consensus-based approach, which was beyond the scope of this study.

### Future Research Directions

A number of practical considerations must be addressed before intraoral scanning can be implemented at scale in epidemiological or public health programs. These include assessing the resource requirements, examiner training needs, and workflow integration within existing service models. Feasibility will also depend on whether the technology is valid and reliable for the specific disease outcomes being evaluated, as different conditions may require tailored scanning protocols or interpretation methods. Additionally, the acceptability, cost-effectiveness, and environmental impact of integrating intraoral scanning into outreach, screening, and teledentistry programs should be evaluated. Research exploring the potential for trained nondental personnel to collect scan data could further support the scalability and sustainability of this approach. Furthermore, investigation into methods that automate caries detection using intraoral scan data is necessary to reduce the time and potential costs associated with using registered dental practitioners for on-screen assessments. This could be achieved through the validation of existing tools [7,34,35] or the development of new artificial intelligence methods [36]. In epidemiological settings, digital data collection combined with automation may potentially improve the frequency of population disease surveillance programs, where data-driven insights into regional oral health trends could be used to inform resource allocation and health promotion efforts.

### Conclusions

This study found that the on-screen assessment of 3D models supplemented with fluorescence substantially agreed with visual examination for caries detection at the moderate and extensive disease thresholds. Whether the on-screen assessment of 3D models generated with IOSs can be used interchangeably with visual examination for caries detection in children is context dependent and likely influenced by the caries threshold used and the tooth surfaces examined. These findings suggest that digital assessments may provide a feasible alternative to traditional in-person examinations for research, longitudinal monitoring, and population-level surveillance, especially where examiner access is limited or standardization across examiners and sites is required.

## Supplementary material

10.2196/78023 Multimedia Appendix 1 Additional tables and figures provided to support the findings reported in this manuscript.

## Abbreviations

HREC: Human Research Ethics Committee
ICC: Intraclass correlation coefficient
ICDAS: International Caries Detection and Assessment System
IOS: intraoral scanner
MIS BAIR: Melbourne Infant Study: BCG for Allergy and Infection Reduction
OR: odds ratio
REDCap: Research Electronic Data Capture
