# Supplementary material for: Dental Caries Detection in Children Using Intraoral Scanners Featuring Fluorescence: Diagnostic Agreement Study
Source: JMIR Public Health Surveill. 2025 Dec 8;11:e78023. doi: 10.2196/78023 (PMC12683709; doi:10.2196/78023)
Supplement: Multimedia Appendix 1 — Additional tables and figures provided to support the findings reported in this manuscript. [file publichealth-v11-e78023-s001.docx]

**Supplementary Appendices**

**Table SA-1:** GRRAS checklist for reporting of studies of reliability and agreement

| **Section** | **Item** | **Checklist Item** | **Reported on Page #** |
| --- | --- | --- | --- |
| **Title/Abstract** | **1** | Identify in title or abstract that interrater/inter-rater  reliability or agreement was investigated | **1** |
| **Introduction** | **2** | Name and describe the diagnostic or measurement device of interest explicitly | **3** |
|  | **3** | Specify the subject population of interest | **4** |
|  | **4** | Specify the rater population of interest (if applicable). |  |
|  | **5** | Describe what is already known about reliability and  agreement and provide a rationale for the study (if  applicable). | **4** |
| **Methods** | **6** | Explain how the sample size was chosen. State the determined number of raters, subjects/objects, and  replicate observations. | No sample size calculation provided.  **6** |
|  | **7** | Describe the sampling method. | **6** |
|  | **8** | Describe the measurement/rating process (e.g. time interval between repeated measurements, availability of clinical information, blinding). | **5-6** |
|  | **9** | State whether measurements/ratings were conducted independently. | **5-6** |
|  | **10** | Describe the statistical analysis. | **6-7** |
| **Results** | **11** | State the actual number of raters and subjects/objects which were included and the number of replicate observations which were conducted. | **8** |
|  | **12** | Describe the sample characteristics of raters and subjects (e.g. training, experience). | **8 and Appendix Table 3** |
|  | **13** | Report estimates of reliability and agreement including measures of statistical uncertainty. | **8-9** |
| **Discussion** | **14** | Discuss the practical relevance of results. | **10-** |
| **Auxiliary Material** | **15** | Provide detailed results if possible (e.g. online). | **Appendices** |

***** Version based on Table I in: Kottner J, Audigé L, Brorson S, Donner A, Gajeweski BJ,

Hróbjartsson A, Robersts C, Shoukri M, Streiner DL. Guidelines for reporting reliability and

agreement studies (GRRAS) were proposed. J Clin Epidemiol. 2011;64(1):96-106

**Table SA-2:** Examiner characteristics, training and calibration results

| Examiner | Scope of practice | Years of experience | Training and calibration exercises | | | | | |
| --- | --- | --- | --- | --- | --- | --- | --- | --- |
|  |  |  | Round 1 (200 teeth) | | | Round 2 (100 teeth) | | |
|  |  |  | Se | Sp | $\kappa$ | Se | Sp | $\kappa$ |
| Examiner 1 | Oral Health Therapist* | 16 | 0.97 | 0.94 | 0.86 | 0.94 | 0.88 | 0.86 |
| Examiner 2 | Oral Health Therapist | 7 | 0.97 | 0.89 | 0.83 | 0.96 | 0.84 | 0.82 |
| Examiner 3 | General Dentist | 10 | 0.89 | 0.98 | 0.77 | 0.86 | 0.96 | 0.75 |
| Examiner 4 | General Dentist** | 3 | 0.80 | 0.92 | 0.80 | 0.85 | 0.92 | 0.83 |

Sensitivity: SE, Specificity: SP Quadratic Weighted Kappa: $\kappa$

*Advanced restorative scope of practice ** Currently enrolled in the Doctor of Clinical Dentistry (paediatric speciality)

**Description of examiner characteristics and on-screen assessment calibration scores**

Examiner 1 (E1), Examiner 2 (E2), Examiner 3 (E3), and Examiner 4 (E4) are all registered dental practitioners and were recruited via convenience sampling. Examiner characteristics are described in Appendix Table 3. E1 and E2 conducted all visual examinations and scanning. Both had prior training and calibration in using ICDAS for research. E1 and E2 were trained for caries assessments using the online training materials and underwent a 2-hour calibration facilitated by an experienced paediatric dentist (MS). Additionally, they underwent training and calibration utilising the EAPD index. After training, both examiners (E1 and E2) participated in separate online scoring exercises for each index, repeating them after two weeks to achieve inter- and intra-rater reliability of >0.8 (weighted kappa) for ICDAS. Four dental practitioners (E1, E2, E3 and E4) conducted all on-screen assessments. They first participated in a 2-hour workshop to learn how to apply a modified ICDAS index to 3D models with fluorescence texture, facilitated by an expert researcher (SM). Examiners then underwent independent calibration to utilise the merged ICDAS criteria on a sample of 3D models with histological reference standards. The first calibration exercises were performed on 200 tooth sites and, after two weeks, repeated on 100 sites to achieve an agreement of >0.7 (quadratic weighted kappa). Examiners were also provided additional training and a standard operating procedure for viewing scans using the TRIOS software and entering data using REDCap.

**
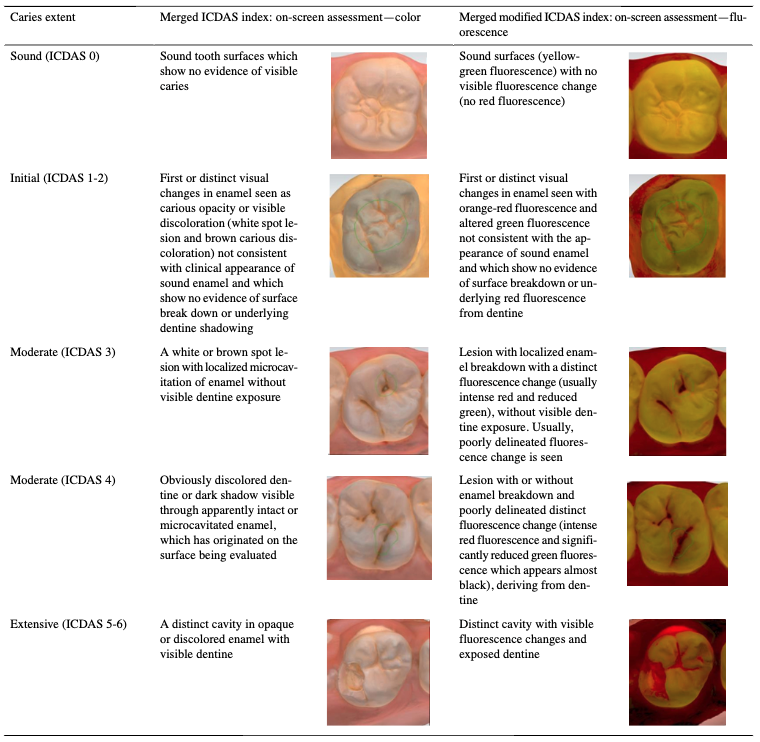
**

**Figure SA-1:** The scoring criteria used for visual examination and on-screen assessment of 3D models, with and without fluorescence, published in Jones, B., Michou, S., Chen, T., Moreno-Betancur, M., Kilpatrick, N., Burgner, D., Vannahme, C., & Silva, M.. (2023). Caries Detection in Primary Teeth Using Intraoral Scanners Featuring Fluorescence: Protocol for a Diagnostic Agreement Study. *JMIR Research Protocols*, *12*, e51578. https://doi.org/10.2196/51578.

**Table SA-3:** Variable Definitions

| Variable | Definition |
| --- | --- |
| Demographic Characteristics | |
| Age | Parent reported date of birth of child. Participant age was calculated as the interval between date of birth and the date of dental assessment, expressed in years and months. Age distribution was summarised using mean and standard deviation. |
| Sex | Parent reported sex of child. Participant sex was recorded as a binary variable, coded as 0 for male and 1 for female participants. It was expressed as a percentage. |
| Socioeconomic status | Socio-economic Indexes for Areas (SEIFA) is a comprehensive Australian metric to describe socioeconomic status. This index combines multiple census-derived parameters including income, education, employment, housing, and family structure to generate geographical rankings of relative socioeconomic disadvantage (Australian Bureau of Statistics, 2021). The Index of Relative Socio-economic Disadvantage can be used to understand an area's relative disadvantage and lack of disadvantage and groups areas (Australian Bureau of Statistics, 2021). The index is published on postal area geographic location, and each participant was assigned a SEIFA score based on their residential postcode. Rankings are split onto deciles, and these were categorised into quintiles for this study, ranging from most disadvantaged (quintile 1) to least disadvantaged (quintile 5) areas. |
| Ethnicity | Parent reported ethnicity of maternal grandmother. Ethnicity was included as was a descriptive categorical variable. It was categorised based on the participant’s maternal grandmother's reported ethnic background, providing a consistent reference point across both cohorts. |
| Education | Parent reported education level. Education level was determined based on the participants mothers' self-reported highest educational attainment. It was included as a descriptive categorical variable. |
| Dental Outcomes and variable definitions | |
|  |  |
| Dental caries class per tooth surface | Primary coronal dental caries per tooth surface. The ICDAS data were combined where necessary to derive four classes: sound (ICDAS 00), initial dental caries (ICDAS 01 and ICDAS 02 lesions), moderate dental caries (ICDAS 03 and ICDAS 04 lesions) and extensive caries (ICDAS 05 and ICDAS 06 lesions). |
| Total caries per person | Patient level outcomes. This variable was created by calculating the total number of primary coronal carious surfaces per person. |
| Presence of enamel defects during visual examination. | The classifications from the modified EAPD index were dichotomised into two classes: enamel defects absent (coded 0) and enamel defects present (coded 1). All enamel defects were combined to derive the enamel defects present variable. |
| Initial disease threshold | A binary variable was created where all sound tooth surfaces were labelled caries absent (code 0), and all remaining classes were labelled caries present (code 1). |
| Moderate disease threshold | A binary variable was created where all sound and initial carious lesions (ICDAS 01 and ICDAS 02) were labelled caries absent (code 0) and all remaining categories were labelled as caries present (1). In the investigation of the automated caries scoring system there was no individual score for extensive caries, so the moderate threshold was referred to as the moderate-extensive threshold in Chapter 5. |
| Extensive disease threshold | A binary variable was created where all sound, initial (ICDAS 01 and ICDAS 02), and moderate classes (ICDAS 03 and ICDAS 04) were combined and labelled as caries absent (code 0) and only the extensive class (ICDAS 05 and ICDAS 06) was labelled as caries present (code 1). |
| Intraoral scanning time | The intraoral scanning time per arch was recorded from the TRIOS scanning software. Intraoral scanning time was summarised in seconds using mean and standard deviation. |
| On-screen assessment time | A time recording field in REDCap was utilised to record the time. This was selected at the start of the on-screen assessment, and then on completion at the on-screen assessment. On-screen time was summarised in minutes and seconds using mean and standard deviation. |

**Table SA-4:** Raw caries scoring data for each method and rater, stratified by surface type.

| **All Surfaces (n=9470)** | | | | | | | | | | | | | | | | | | | | | | | | | | | |
| --- | --- | --- | --- | --- | --- | --- | --- | --- | --- | --- | --- | --- | --- | --- | --- | --- | --- | --- | --- | --- | --- | --- | --- | --- | --- | --- | --- |
| **Score** | **VE** | **OSA** | **OSA-FLU** | **On-Screen assessment without fluorescence** | | | | | | | | | | | | **On-Screen assessment with fluorescence** | | | | | | | | | | | |
|  |  |  |  | **Rater 1** | | | **Rater 2** | | | **Rater 3** | | | **Rater 4** | | | **Rater 1** | | | **Rater 2** | | | **Rater 3** | | | **Rater 4** | | |
|  |  |  |  | **T1** | **T2** | **x̄** | **T1** | **T2** | **x̄** | **T1** | **T2** | **x̄** | **T1** | **T2** | **x̄** | **T1** | **T2** | **x̄** | **T1** | **T2** | **x̄** | **T1** | **T2** | **x̄** | **T1** | **T2** | **x̄** |
| Sound | 9,182 | 9,154 | 9,125 | 9,247 | 9,213 | 9230 | 9,154 | 9,181 | 9,168 | 9,187 | 9,177 | 9182 | 9,015 | 9,055 | 9035 | 9,149 | 9,164 | 9156.5 | 9,127 | 9,140 | 9133.5 | 9,159 | 9,179 | 9169 | 9,017 | 9,065 | 9041 |
| Initial Caries | 202 | 241 | 269 | 149 | 179 | 164 | 225 | 204 | 215 | 214 | 222 | 218 | 387 | 351 | 369 | 245 | 225 | 235 | 248 | 242 | 245 | 240 | 223 | 231.5 | 387 | 341 | 364 |
| Moderate Caries- Micro cavitation | 7 | 12.125 | 11.625 | 7 | 12 | 9.5 | 12 | 11 | 12 | 12 | 17 | 14.5 | 14 | 12 | 13 | 5 | 10 | 7.5 | 14 | 12 | 13 | 13 | 14 | 13.5 | 14 | 11 | 12.5 |
| Moderate Caries- Shadow | 31 | 16.625 | 19.75 | 18 | 18 | 18 | 25 | 24 | 25 | 11 | 7 | 9 | 18 | 12 | 15 | 27 | 25 | 26 | 28 | 24 | 26 | 12 | 11 | 11.5 | 18 | 13 | 15.5 |
| Extensive Caries | 48 | 46.25 | 44.75 | 49 | 48 | 48.5 | 54 | 50 | 52 | 46 | 47 | 46.5 | 36 | 40 | 38 | 44 | 46 | 45 | 53 | 52 | 52.5 | 46 | 43 | 44.5 | 34 | 40 | 37 |
| **Occlusal Surfaces (n=1602)** | | | | | | | | | | | | | | | | | | | | | | | | | | | |
| **Score** | **VE** | **OSA** | **OSA-FLU** | **On-Screen assessment without fluorescence** | | | | | | | | | | | | **On-Screen assessment with fluorescence** | | | | | | | | | | | |
|  |  |  |  | **Rater 1** | | | **Rater 2** | | | **Rater 3** | | | **Rater 4** | | | **Rater 1** | | | **Rater 2** | | | **Rater 3** | | | **Rater 4** | | |
|  |  |  |  | **T1** | **T2** | **x̄** | **T1** | **T2** | **x̄** | **T1** | **T2** | **x̄** | **T1** | **T2** | **x̄** | **T1** | **T2** | **x̄** | **T1** | **T2** | **x̄** | **T1** | **T2** | **x̄** | **T1** | **T2** | **x̄** |
| Sound | 1,438 | 1,401 | 1,372 | 1,462 | 1,453 | 1,458 | 1,413 | 1,419 | 1,416 | 1,425 | 1,410 | 1,418 | 1,304 | 1,321 | 1,313 | 1,389 | 1,411 | 1,400 | 1,384 | 1,381 | 1382.5 | 1,394 | 1,405 | 1399.5 | 1,295 | 1,318 | 1306.5 |
| Initial Caries | 121 | 163 | 189 | 102 | 110 | 106 | 146 | 138 | 142 | 141 | 155 | 148 | 262 | 247 | 254.5 | 170 | 147 | 158.5 | 171 | 173 | 172 | 172 | 159 | 165.5 | 271 | 249 | 260 |
| Moderate Caries- Micro cavitation | 2 | 5.25 | 4.5 | 2 | 1 | 2 | 5 | 2 | 3.5 | 7 | 11 | 9 | 7 | 7 | 7 | 0 | 0 | 0 | 5 | 3 | 4 | 5 | 9 | 7 | 7 | 7 | 7 |
| Moderate Caries- Shadow | 21 | 12.625 | 15.75 | 15 | 18 | 17 | 11 | 20 | 15.5 | 7 | 6 | 6.5 | 14 | 10 | 12 | 22 | 25 | 23.5 | 15 | 20 | 17.5 | 9 | 9 | 9 | 15 | 11 | 13 |
| Extensive Caries | 20 | 20.625 | 20.625 | 21 | 20 | 21 | 27 | 23 | 25 | 22 | 20 | 21 | 15 | 17 | 16 | 21 | 19 | 20 | 27 | 25 | 26 | 22 | 20 | 21 | 14 | 17 | 15.5 |
| **Smooth Surfaces (n= 7868)** | | | | | | | | | | | | | | | | | | | | | | | | | | | |
| **Score** | **VE** | **OSA** | **OSA-FLU** | **On-Screen assessment without fluorescence** | | | | | | | | | | | | **On-Screen assessment with fluorescence** | | | | | | | | | | | |
|  |  |  |  | **Rater 1** | | | **Rater 2** | | | **Rater 3** | | | **Rater 4** | | | **Rater 1** | | | **Rater 2** | | | **Rater 3** | | | **Rater 4** | | |
|  |  |  |  | **T1** | **T2** | **x̄** | **T1** | **T2** | **x̄** | **T1** | **T2** | **x̄** | **T1** | **T2** | **x̄** | **T1** | **T2** | **x̄** | **T1** | **T2** | **x̄** | **T1** | **T2** | **x̄** | **T1** | **T2** | **x̄** |
| Sound | 7,744 | 7,753 | 7,753 | 7,785 | 7,760 | 7772.5 | 7,741 | 7,762 | 7751.5 | 7,762 | 7,767 | 7764.5 | 7,711 | 7,734 | 7722.5 | 7,760 | 7,753 | 7756.5 | 7,743 | 7,759 | 7751 | 7,765 | 7,774 | 7769.5 | 7,722 | 7,747 | 7734.5 |
| Initial Caries | 81 | 78.75 | 79.875 | 47 | 69 | 58 | 79 | 66 | 72.5 | 73 | 67 | 70 | 125 | 104 | 114.5 | 75 | 78 | 76.5 | 77 | 69 | 73 | 68 | 64 | 66 | 116 | 92 | 104 |
| Moderate Caries- Micro cavitation | 5 | 6.875 | 7.125 | 5 | 11 | 8 | 7 | 9 | 8 | 5 | 6 | 5.5 | 7 | 5 | 6 | 5 | 10 | 7.5 | 9 | 9 | 9 | 8 | 5 | 6.5 | 7 | 4 | 5.5 |
| Moderate Caries- Shadow | 10 | 4 | 4 | 3 | 0 | 1.5 | 14 | 4 | 9 | 4 | 1 | 2.5 | 4 | 2 | 3 | 5 | 0 | 2.5 | 13 | 4 | 8.5 | 3 | 2 | 2.5 | 3 | 2 | 2.5 |
| Extensive Caries | 28 | 25.625 | 24.125 | 28 | 28 | 28 | 27 | 27 | 27 | 24 | 27 | 25.5 | 21 | 23 | 22 | 23 | 27 | 25 | 26 | 27 | 26.5 | 24 | 23 | 23.5 | 20 | 23 | 21.5 |

VE: Visual examination, OSA: On-screen assessment in colour only, OSA-FLU: On-screen assessment with fluorescence

**Table SA-5:** Scoring data for each method and rater by disease threshold. Stratified by surface type.

| Disease Threshold | Visual Examination | | On-screen assessment with colour | | | | | | | | | | | | | | | | | |
| --- | --- | --- | --- | --- | --- | --- | --- | --- | --- | --- | --- | --- | --- | --- | --- | --- | --- | --- | --- | --- |
|  | No | Yes | Average Total | | Rater 1 | | | | Rater 2 | | | | Rater 3 | | | | Rater 4 | | | |
|  |  |  | No | Yes | T1 | | T2 | | T1 | | T2 | | T1 | | T2 | | T1 | | T2 | |
|  |  |  |  |  | No | Yes | No | Yes | No | Yes | No | Yes | No | Yes | No | Yes | No | Yes | No | Yes |
| Initial | 9182 | 288 | 9154 | 316 | 9,247 | 223 | 9,213 | 257 | 9,154 | 316 | 9,181 | 289 | 9,187 | 283 | 9,177 | 293 | 9,015 | 455 | 9,055 | 415 |
| Moderate | 9384 | 86 | 9395 | 75 | 9,396 | 74 | 9,392 | 78 | 9,379 | 91 | 9,385 | 85 | 9,401 | 69 | 9,399 | 71 | 9,402 | 68 | 9,406 | 64 |
| Extensive | 9422 | 48 | 9424 | 46 | 9,421 | 49 | 9,422 | 48 | 9,416 | 54 | 9,420 | 50 | 9,424 | 46 | 9,423 | 47 | 9,434 | 36 | 9,430 | 40 |
| Disease Threshold | Visual Examination | | On-Screen assessment with fluorescence | | | | | | | | | | | | | | | | | |
|  | No | Yes | Average Total | | Rater 1 | | | | Rater 2 | | | | Rater 3 | | | | Rater 4 | | | |
|  |  |  | No | Yes | T1 | | T2 | | T1 | | T2 | | T1 | | T2 | | T1 | | T2 | |
|  |  |  |  |  | No | Yes | No | Yes | No | Yes | No | Yes | No | Yes | No | Yes | No | Yes | No | Yes |
| Initial | 9182 | 288 | 9125 | 345 | 9,149 | 321 | 9,164 | 306 | 9,127 | 343 | 9,140 | 330 | 9,159 | 311 | 9,179 | 291 | 9,017 | 453 | 9,065 | 405 |
| Moderate | 9384 | 86 | 9394 | 76 | 9,394 | 76 | 9,389 | 81 | 9,375 | 95 | 9,382 | 88 | 9,399 | 71 | 9,402 | 68 | 9,404 | 66 | 9,406 | 64 |
| Extensive | 9422 | 48 | 9380 | 45 | 9,426 | 44 | 9,424 | 46 | 9,417 | 53 | 9,418 | 52 | 9,424 | 46 | 9,427 | 43 | 9,436 | 34 | 9,430 | 40 |
| Disease Threshold | Visual Examination | | On-screen assessment with colour | | | | | | | | | | | | | | | | | |
|  | No | Yes | Average Total | | Rater 1 | | | | Rater 2 | | | | Rater 3 | | | | Rater 4 | | | |
|  |  |  | No | Yes | T1 | | T2 | | T1 | | T2 | | T1 | | T2 | | T1 | | T2 | |
|  |  |  |  |  | Yes | No | Yes | No | Yes | No | Yes | No | Yes | No | Yes | No | Yes | No | Yes | No |
| Initial | 1438 | 164 | 1401 | 201 | 1,462 | 140 | 1,453 | 149 | 1,413 | 189 | 1,419 | 183 | 1,425 | 177 | 1,410 | 192 | 1,304 | 298 | 1,321 | 281 |
| Moderate | 1559 | 43 | 1564 | 39 | 1,564 | 38 | 1,563 | 39 | 1,559 | 43 | 1,557 | 45 | 1,566 | 36 | 1,565 | 37 | 1,566 | 36 | 1,568 | 34 |
| Extensive | 1582 | 20 | 1581 | 21 | 1,581 | 21 | 1,582 | 20 | 1,575 | 27 | 1,579 | 23 | 1,580 | 22 | 1,582 | 20 | 1,587 | 15 | 1,585 | 17 |
| Disease Threshold | Visual Examination | | On-Screen assessment with fluorescence | | | | | | | | | | | | | | | | | |
|  | No | Yes | Average Total | | Rater 1 | | | | Rater 2 | | | | Rater 3 | | | | Rater 4 | | | |
|  |  |  | No | Yes | T1 | | T2 | | T1 | | T2 | | T1 | | T2 | | T1 | | T2 | |
|  |  |  |  |  | No | Yes | No | Yes | No | Yes | No | Yes | No | Yes | No | Yes | No | Yes | No | Yes |
| Initial | 1438 | 164 | 1372 | 230 | 1,389 | 213 | 1,411 | 191 | 1,384 | 218 | 1,381 | 221 | 1,394 | 208 | 1,405 | 197 | 1,295 | 307 | 1,318 | 284 |
| Moderate | 1559 | 43 | 1561 | 41 | 1,559 | 43 | 1,558 | 44 | 1,555 | 47 | 1,554 | 48 | 1,566 | 36 | 1,564 | 38 | 1,566 | 36 | 1,567 | 35 |
| Extensive | 1582 | 20 | 1581 | 21 | 1,581 | 21 | 1,583 | 19 | 1,575 | 27 | 1577.0 | 25.0 | 1,580 | 22 | 1,582 | 20 | 1,588 | 14 | 1,585 | 17 |
| Disease Threshold | Visual Examination | | On-screen assessment with color | | | | | | | | | | | | | | | | | |
|  | No | Yes | Average Total | | Rater 1 | | | | Rater 2 | | | | Rater 3 | | | | Rater 4 | | | |
|  |  |  | No | Yes | T1 | | T2 | | T1 | | T2 | | T1 | | T2 | | T1 | | T2 | |
|  |  |  |  |  | Yes | No | Yes | No | Yes | No | Yes | No | Yes | No | Yes | No | Yes | No | Yes | No |
| Initial | 7744 | 124 | 7753 | 115 | 7,785 | 83 | 7,760 | 108 | 7,741 | 127 | 7,762 | 106 | 7,762 | 106 | 7,767 | 101 | 7,711 | 157 | 7,734 | 134 |
| Moderate | 7825 | 43 | 7832 | 37 | 7,832 | 36 | 7,829 | 39 | 7,820 | 48 | 7,828 | 40 | 7,835 | 33 | 7,834 | 34 | 7,836 | 32 | 7,838 | 30 |
| Extensive | 7840 | 28 | 7842 | 26 | 7,840 | 28 | 7,840 | 28 | 7,841 | 27 | 7,841 | 27 | 7,844 | 24 | 7,841 | 27 | 7,847 | 21 | 7,845 | 23 |
| Disease Threshold | Visual Examination | | On-Screen assessment with fluorescence | | | | | | | | | | | | | | | | | |
|  | No | Yes | Average Total | | Rater 1 | | | | Rater 2 | | | | Rater 3 | | | | Rater 4 | | | |
|  |  |  | No | Yes | T1 | | T2 | | T1 | | T2 | | T1 | | T2 | | T1 | | T2 | |
|  |  |  |  |  | No | Yes | No | Yes | No | Yes | No | Yes | No | Yes | No | Yes | No | Yes | No | Yes |
| Initial | 7744 | 124 | 7753 | 115 | 7,760 | 108 | 7,753 | 115 | 7,743 | 125 | 7,759 | 109 | 7,765 | 103 | 7,774 | 94 | 7,722 | 146 | 7,747 | 121 |
| Moderate | 7825 | 43 | 7833 | 37 | 7,835 | 33 | 7,831 | 37 | 7,820 | 48 | 7,828 | 40 | 7,833 | 35 | 7,838 | 30 | 7,838 | 30 | 7,839 | 29 |
| Extensive | 7840 | 28 | 7844 | 26 | 7,845 | 23 | 7,841 | 27 | 7,842 | 26 | 7841.0 | 27.0 | 7,844 | 24 | 7,845 | 23 | 7,848 | 20 | 7,845 | 23 |
|  |  |  |  |  |  |  |  |  |  |  |  |  |  |  |  |  |  |  |  |  |

**Table SA-6-: Diagnostic Agreement between methods by surface type.** Agreement estimates for visual examination (VE) versus. on-screen assessment (OSA), and VE versus. on-screen assessment with fluorescence (OSA-FLU). The odds ratios (ORs) and 95% Confidence Intervals (CI) are shown, stratified by surface type. The main analysis, which contained the variable Method as a fixed effect, is shown, in addition to the sensitivity analysis, where enamel defects at VE was fit as an additional co-variate in the multilevel model.

| **Occlusal surfaces ( n=1,602)** | | | | | | | | | | | | |
| --- | --- | --- | --- | --- | --- | --- | --- | --- | --- | --- | --- | --- |
| **Disease threshold** | **VE** | | **OSA** | | **Main analysis** | | | | **Sensitivity analysis** | | | |
|  | **No** | **Yes** | **No** | **Yes** | **OR (95% CI)** | | | ***P*** | **OR (95% CI)** | | | ***P*** |
| Initial threshold | 1438 | 164 | 1401 | 201 | 1.5 | (1.2 | 1.9) | <0.001 | 1.4 | (1.2 | 1.8) | <0.001 |
| Moderate threshold | 1559 | 43 | 1564 | 39 | 0.9 | (0.6 | 1.3) | 0.41 | 0.8 | (0.6 | 1.2) | 0.37 |
| Extensive threshold | 1582 | 20 | 1581 | 21 | 1.0 | (0.6 | 1.8) | 0.88 | 1.0 | (0.6 | 1.8) | 0.90 |
| **Disease threshold** | **VE** | | **OSA- FLU** | | **Main analysis** | | | | **Sensitivity analysis** | | | |
|  | **No** | **Yes** | **No** | **Yes** | **OR (95% CI)** | | | ***P*** | **OR (95% CI)** | | | ***P*** |
| Initial threshold | 1438 | 164 | 1372 | 230 | 2.0 | (1.6 | 2.4) | <0.001 | 1.9 | (1.5 | 2.4) | <0.001 |
| Moderate threshold | 1559 | 43 | 1561 | 41 | 0.9 | (0.6 | 1.4) | 0.70 | 0.9 | (0.6 | 1.2) | 0.65 |
| Extensive threshold | 1582 | 20 | 1581 | 21 | 1.0 | (0.6 | 1.8) | 0.88 | 1.0 | (0.6 | 1.8) | 0.90 |
| **Smooth surfaces (n=7,868)** | | | | | | | | | | | | |
| **Disease threshold** | **VE** | | **OSA** | | **Main analysis** | | | | **Sensitivity analysis** | | | |
|  | **No** | **Yes** | **No** | **Yes** | **OR (95% CI)** | | | ***P*** | **OR (95% CI)** | | | ***P*** |
| Initial threshold | 7744 | 124 | 7753 | 115 | 0.9 | (0.7 | 1.1) | 0.37 | 0.9 | (0.7 | 1.1) | 0.22 |
| Moderate threshold | 7825 | 43 | 7832 | 37 | 0.8 | (0.5 | 1.2) | 0.23 | 0.8 | (0.5 | 1.2) | 0.22 |
| Extensive threshold | 7840 | 28 | 7842 | 26 | 0.9 | (0.5 | 1.5) | 0.56 | 0.9 | (0.5 | 1.4) | 0.55 |
| **Disease threshold** | **VE** | | **OSA- FLU** | | **Main analysis** | | | | **Sensitivity analysis** | | | |
|  | **No** | **Yes** | **No** | **Yes** | **OR (95% CI)** | | | ***P*** | **OR (95% CI)** | | | ***P*** |
| Initial threshold | 7744 | 124 | 7753 | 115 | 0.9 | (0.7 | 1.1) | 0.37 | 0.9 | (0.7 | 1.1) | 0.21 |
| Moderate threshold | 7825 | 43 | 7833 | 37 | 0.8 | (0.5 | 1.1) | 0.15 | 0.7 | (0.5 | 1.1) | 0.14 |
| Extensive threshold | 7840 | 28 | 7844 | 26 | 0.8 | (0.5 | 1.3) | 0.33 | 0.8 | (0.5 | 1.3) | 0.32 |

**
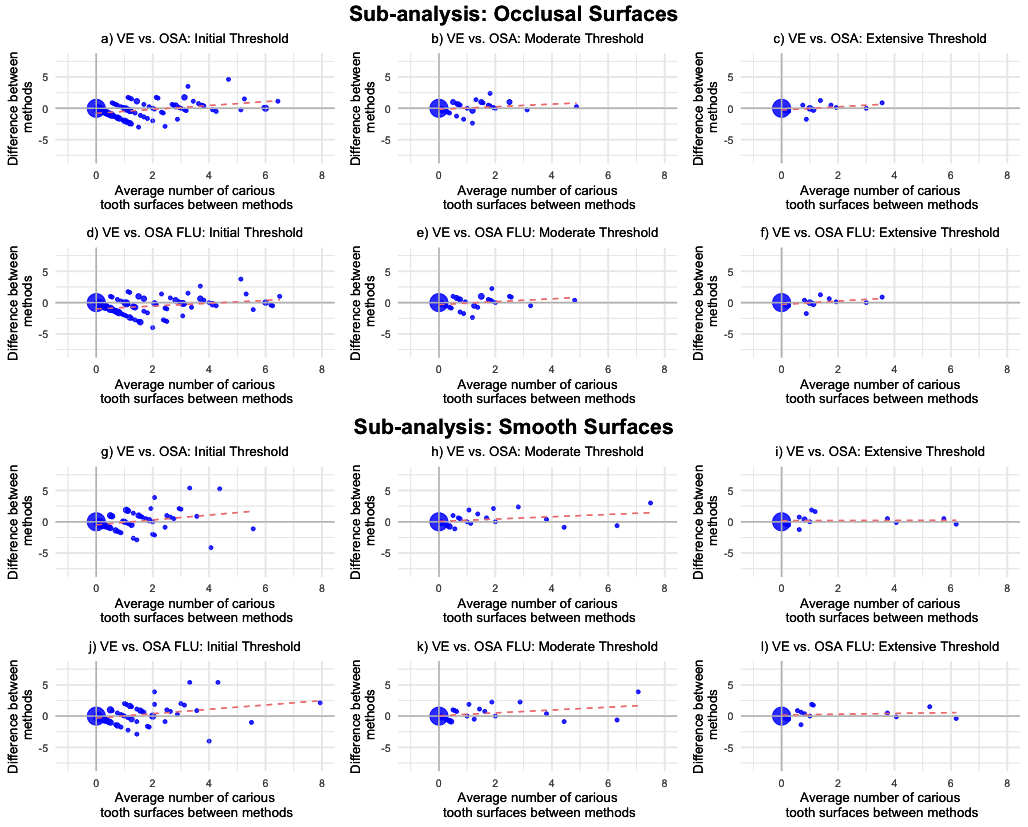
**

**Figure SA-2:** Bland Altman graphs: Sub-analysis by surface type.

Bland Altman graphs comparing the differences in the number of carious lesions detected for each pair of methods against the average of the two methods for the initial, moderate and extensive disease thresholds for occlusal surfaces and smooth surfaces. The regression line of the differences in methods against the mean is represented by a red dash. The size of the data points is proportional to the number of observations. VE: Visual Examination, OSA: On-screen Assessment in colour only, OSA FLU: On-screen Assessment with Fluorescence.
